# Genetic Changes Driving Immunosuppressive Microenvironments in Oral Premalignancy

**DOI:** 10.3389/fimmu.2022.840923

**Published:** 2022-01-27

**Authors:** Roberto Rangel, Curtis R. Pickering, Andrew G. Sikora, Michael T. Spiotto

**Affiliations:** ^1^ Department of Head and Neck Surgery, The University of Texas M.D. Anderson Cancer Center, Houston, TX, United States; ^2^ Department of Radiation Oncology, The University of Texas M.D. Anderson Cancer Center, Houston, TX, United States

**Keywords:** head and neck cancer, immunosuppression, TP53, CDKN2A, NOTCH1

## Abstract

Oral premalignant lesions (OPLs) are the precursors to oral cavity cancers, and have variable rates of progression to invasive disease. As an intermediate state, OPLs have acquired a subset of the genomic alterations while arising in an oral inflammatory environment. These specific genomic changes may facilitate the transition to an immune microenvironment that permits malignant transformation. Here, we will discuss mechanisms by which OPLs develop an immunosuppressive microenvironment that facilitates progression to invasive cancer. We will describe how genomic alterations and immune microenvironmental changes co-evolve and cooperate to promote OSCC progression. Finally, we will describe how these immune microenvironmental changes provide specific and unique evolutionary vulnerabilities for targeted therapies. Therefore, understanding the genomic changes that drive immunosuppressive microenvironments may eventually translate into novel biomarker and/or therapeutic approaches to limit the progression of OPLs to potential lethal oral cancers.

## Introduction

Oral squamous cell carcinomas (OSCCs) involving the tongue, cheek, gums, and other sites of the mouth are the most common form of head and neck cancer (HNSCC), responsible for over 377,000 new cases and 177,000 deaths per year worldwide (1). Up to 50% of people developing OSCCs die from this disease. Even with cure, OSCCs are often treated with surgery, chemotherapy and radiation leading to substantial adverse impact on cosmesis, function, and quality of life.

The precursors to OSCC are oral premalignant lesions (OPLs) that affect approximately 4.5% of the world’s population (2). Another commonly used term for these lesions is oral potentially malignant disorders (OPMD). Because we are focusing on the progression of these lesions to cancer in both human and mouse systems we will use the term OPL throughout this review. OPLs include a variety of distinct pathological entities including leukoplakia, submucous fibrosis, and lichen planus. OPL remains a diagnostic dilemma with limited preventative and therapeutic options. Even though the majority of OPLs regress, up to 30% of OPL ultimately progress through increasingly severe grades of dysplasia to oral cancer. The overall annual risk of transformation of leukoplakia (the most common variety in the US) to invasive cancer is 1- 3% per year (3). Since OPLs often present with multifocal lesions across the oral mucosa, patients are subjected to frequent biopsies until a cancer is detected and is surgically removed. At present, diagnosis of OPL requires a physical biopsy in order to distinguish OPL from early OSCC. Consequently, clinicians have few if any noninvasive biomarkers to predict which OPL are at high risk of progression to invasive cancer (4). Furthermore, the only existing treatment for OPL is excision of the lesion with a margin of surrounding healthy tissue. Nevertheless, it is often not practical to remove the entire OPL because OPLs may encompass a large region of the oral cavity while still at variable risk for progressing to invasive disease. Consequently, the diagnostic and therapeutic dilemmas in treating OPLs provide impetus to better understand the biological and immunological underpinnings of this disease.

Representing an intermediate phase during the evolution of normal mucosa into malignant cancer, OPLs have acquired only a subset of the genomic alterations necessary to develop into OSCC. In addition to genomic changes occurring in OPL, OPLs frequently arise in an oral inflammatory environment caused by exogenous factors such tobacco and/or alcohol use, poor dentition and by endogenous factors including auto-immune diseases. It is likely that the genomic alterations cooperate and co-regulate the immune microenvironment in OPLs to facilitate the progression of OPLs to invasive cancer (1, 5). Namely, specific genomic changes may facilitate the transition to an immune microenvironment that permits malignant transformation. Conversely, inflammatory changes in the immune microenvironment may promote genomic instability within OPLs. Here, we will discuss the how OPLs develop an immunosuppressive microenvironment that facilitates progression to invasive cancer. We will then describe how genomic alterations and immune microenvironmental changes co-evolve and cooperate to promote OSCC progression. Finally, we will describe how the genomic context of premalignant lesions may provide specific and unique evolutionary vulnerabilities for targeted therapies. Pursuit of such paradigms may eventually translate into novel biomarker and/or therapeutic use.

## The Evolution of the Immunosuppressive Microenvironment in OPL

**Figure 1:**
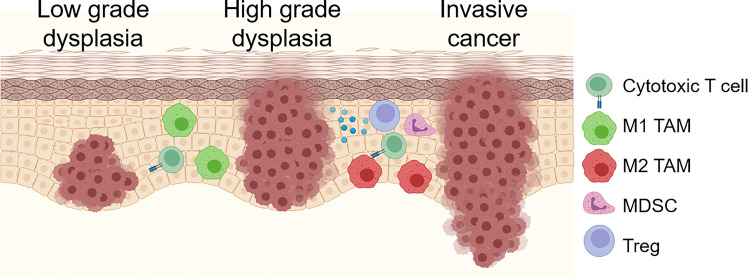
Schema for immune/inflammatory microenvironment evolution during OPL progression. OPL are often strongly infiltrated with CD8^+^ T cells. Early low grade dysplasias also demonstrate tumoricidal and/or M1 TAMs. As dysplastic lesions progress, increased immunosuppressive M2 TAM, Treg, MDSCs and immunosuppressive molecules including PD-1/PD-L1 and A2AR. Created with BioRender.com.

As mutations and other genetic alterations accumulate, OPL become progressively infiltrated with immune suppressive myeloid cells including MDSC, M2 tumor-associated macrophages (TAM) and regulatory T cells (Treg; **Figure 1**). Furthermore, M2 macrophage polarization, Treg infiltration, and expression of the immune checkpoint ligand PD-L1 are associated with increased risk of future malignant transformation (6–9). Despite this progressive accumulation of immune suppressive features, OPL are often strongly infiltrated with CD8^+^ T lymphocytes (10, 11), suggesting that reversing the immunosuppressive microenvironment of high-risk OPL has the potential to unmask anti-lesion immunity capable of inducing immune regression prior to development of invasive cancer. Here, we will describe the inflammatory and immune changes that occur during OPL progression as well as possible genetic mechanisms drive the cross-talk between the genomic and immune changes.

The transition from OPL to invasive cancer requires that dysplastic lesions escape recognition from infiltrating cytotoxic T lymphocytes (TILs). TILs are increased in OPLs and predict histological grade in dysplastic lesions (12–14). Comparing lichen planus with oral dysplastic lesions, Flores-Hidalgo et al. demonstrated that CD8^+^ T cells tracked the malignant transformation zones in dysplastic lesions (15). Similarly, Gannot et al. demonstrated increased CD4^+^, CD8^+^ and CD19^+^/CD20^+^ lymphocyte infiltration in dysplastic lesions compared to normal epithelium indicating that dysplastic lesions are accompanied by increased cytotoxic and helper T cells as well as B cells (16). Since these studies only quantified infiltrating lymphocytes, it remains unclear if these TILs are functional and target the dysplastic epithelial cells or more indicative of the general inflammatory microenvironment. However, other reports showed infiltrating plasms cells and other B lymphocytes decreased with high grades of dysplasia and less differentiated OSCCs (17). Similarly, in a 4-nitroquinoline 1-oxide (4-NQO) chemical carcinogenesis mouse model, lymphocytic infiltration was associated with dysplastic and invasive lesions (18).

The role of CD8+ T cell infiltrating OPLs remain unclear. Similar to invasive disease, it remains unclear what antigens these cytotoxic lymphocytes recognize. Over 40% of progressing OPLs expressed at least one of the shared MAGE cancer testis antigens (19). Similarly, common shared antigens such as NY-ESO-1 and MUC1 have been observed in murine OPL (20). Although on average OPL has 167,809 variants per sample, there did not appear to be any differences in mutational burdens between progressing and non-progressing premalignant lesions using a small series of 13 patients (21). Furthermore, the role of neo-antigens form by somatic mutations as targets for infiltrating lymphocytes in OPL remains understudied. Finally, some of these TILs may represent bystander T cells that do not recognize cognate antigens in OPL as seen in other cancers (22). Therefore, OPL does express tumor antigens but the specificities of the TILs remain understudied.

Given the increased lymphocytic infiltration observed in OPLs and OSCCs, immunosuppressive mechanisms are likely required to facilitate the progression of normal epithelium to dysplasia and invasive cancer. To this end, Zhao et al. demonstrated increased regulatory T cells (Tregs), as measured by CD4^+^CD25^+^FoxP3^+^ markers, were sequentially increased in the lymph nodes and in the peripheral blood of rats with worsening grades of dysplasia and OSCC (23). Similarly, increased Tregs and decreased IFNγ signaling were observed in the development of oral leukoplakia, oral lichen planus and OSCCs (24). Immunosuppressive molecules may also prevent infiltrating lymphocytes from clearing pre-malignant lesions. A meta-analysis of 9 studies demonstrated that PD-L1 may be enriched in OPL as 48.25% of lesions expressed PD-L1 which was on average 1.65-fold greater than normal mucosa (25). PD-L1 expression in both epithelial cells and tumor associated macrophages (TAMs) was also correlated with malignant transformation (7). In a 4-NQO model of chemical carcinogenesis, Chen et al. observed a nearly 2-fold increase in PD-1 expression on infiltrating CD8^+^ and CD4^+^ T cells. PD-1 inhibition reduced MDSCs as well as downregulated PD-1 on TIL that was associated with a 2.27-fold increase in activated TIL (26). Finally, upregulation of the adenosine receptor (A2AR) also likely suppresses anti-tumor lymphocytes as A2AR expression correlated with pathological grade, lymph node status (27). Consequently, OPLs may recruit immunosuppressive Tregs and/or express immunosuppressive checkpoint molecules such as PD-L1 to evade potential rejection by cytotoxic lymphocytes.

Oral dysplastic lesions may also recruit immunosuppressive myeloid cells to evade immune recognition. In many types of solid tumors, TAMs and MDSCs correlated with poor prognosis and progression to invasive cancers (12, 16, 28–31). OPL progression has been associated with increased MDSC and TAM infiltration as well as the polarization of TAM from so-called M1 (tumor-suppressing) to M2 (tumor-promoting) phenotypes in murine models and human cancers. Both pro-tumorigenic M2 tumor associated macrophages (TAM) and myeloid-derived suppressor cells (MDSC) may be a key regulator for governing these pro-tumorigenic and anti-tumorigenic immune responses in OPL by suppressing adaptive T cell immunity and by producing inflammatory cytokines to promote tumor proliferation and angiogenesis (24). Abnormal proliferation of epithelial cells may promote TAM and myeloid cell infiltration. Kawsar et al. demonstrated that human beta-defensin 3 (hBD-3) but not hBD-1 or hBD-2 colocalized to proliferating basal cells in normal epithelium as well as in dysplastic lesions. Furthermore, the increased hBD-3 in dysplastic lesions increased macrophage recruitment in oral dysplastic lesions as well as increased macrophage chemotaxis *in vitro* (32). Consequently, epithelial proliferation that is intrinsic in OPL recruit immunosuppressive myeloid subsets.

Paradoxically, TAMs in OPLs have been shown to display both tumoricidal macrophages and tumorigenic functions. In 58 OPLs and 258 OSCCs, Wang et al. demonstrated that increase CD163^+^ M2 TAMs were associated with OSCC progression and survival (33). Similarly, Kouketsu et al. found increasing numbers of M2 TAMs, using both the CD163 and CD204 markers for the M2 phenotype, and regulatory T cells with higher grades of OPL (8). Yagyuu et al. demonstrated that dermal M2 macrophages, identified by the CD163 marker, correlated with increased dysplasia (7). However, some groups indicate that TAMs may inhibit OPL regardless of M1 or M2 phenotype. Bouaoud et al. used immune cell deconvolution and enrichment algorithms for RNAseq as well as immunohistochemistry on OPL to observe that M2 macrophages were associated with dysplasia and OSCC even though 3 M2 TAM signatures were associated with better oral cancer-free survival (34). By contrast, Mori et al. demonstrated that the M2 canonical marker CD163 may identify TAMs with an inflammatory rather than immunosuppressive phenotype. CD163^+^ TAMs in OPLs displayed increased STAT1 and CXCL9 expression suggesting an inflammatory tumoricidal phenotype (31). The authors stipulated that this M1 phenotype was likely driven by TH1 CD4^+^ T cells producing IFN to drive this anti-tumorigenic phenotype (35). This contrasts with other reports demonstrating that CD163^+^ TAMs in oral dysplasia expressing immune suppressive cytokines such as IL-10 (36). One possibility to rectify these observations is that the TAM phenotype transitions from a tumoricidal M1-like phenotype to a tumorigenic M2-like phenotype during OPL progression. In 201 OPL specimens, Weber et al. examined both M1 and M2 TAM phenotypes using the canonical markers CD68 and CD163, respectively (37). Increased TAM infiltration, TAM localization to the epithelial compartment and M2 polarization was associated with progression of OPL to invasive cancer. Therefore, TAM phenotypes may represent a dynamic state as OPLs progress to invasive lesions.

Several environmental and/or immune changes may dictate the dynamic changes in TAM phenotype during OPL progression. One potential mediator linking this immune switch during OPL progression is inducible nitric oxide synthase (iNOS), which recruits MDSCs and TAMs as well as polarizes TAMs to the pro-tumorigenic M2 subtype (38–40). iNOS has multiple pleiotropic and contradictory roles in promotion and suppression of cancer development. iNOS is a well-described driver of oncogenic signaling and immune evasion mechanisms in established cancers.

However, iNOS can also act as a mechanism of M1 macrophage anti-tumor activity, and when expressed in CD4^+^ T cells can inhibit their differentiation to Treg (41) or Th17 cells (42). These contradictory roles for iNOS highlight the importance of assessing iNOS function in a cell type and context specific manner. iNOS expression and immune suppressive myeloid populations (M2 macrophages and MDSC) have both been shown to increase during progressive stages of dysplastic transformation and to be associated with future risk of transition to invasive cancer. iNOS can play different pro-tumor and anti-tumor roles depending on timing of expression and in which cell types. iNOS is known to be expressed by M1 (anti-tumor) TAM where it exerts a tumoricidal function through inducing tumor cytolysis. We have also shown that iNOS expression in myeloid cells acts paradoxically as a negative feedback mechanism to suppresses M1 macrophage polarization (43). However, iNOS (along with Arginase and PD-L1 expression) is a major immune suppressive mechanism of MDSC. We have also shown an important role for tumor-expressed iNOS in orchestrating the induction of tumor-infiltrating myeloid cells and acquisition of MDSC suppressive function in established cancer (39, 44). The apparent paradoxical pleiotropy and cell type specificity of iNOS expression and function highlights the importance of distinguishing between tumor- and myeloid cell-expressed iNOS functions.

In addition, linking environmental risk factors with TAM phenotypes in OPLs, Zhu et al. demonstrated that cigarette smoke extract increased M2 macrophage OPL infiltration and polarization, increased immunosuppressive cytokines including arginase-1 and IL-10 and decreased pro-inflammatory markers TNFα and iNOS (45). Furthermore, this group proposed that smoking activated glutamine transport and metabolism in TAM to promote epithelial proliferation and inhibit apoptosis.

Myeloid derived suppressor cells (MDSCs) which have been shown inhibit anti-tumor immunity also correlate with OPL progression. Both CD33^+^ tumor infiltrating and circulating MDSC are increased in oral leukoplakia patients with increasingly severe dysplasia (9, 46). In a chemical carcinogenesis model of OPL, P. gingivalis colonization, common in oral cancer patients, further increased MDSC accumulation which was associated with increases in the chemokines CXCL2 and CCL2 as well as the cytokines IL-6 and IL-8 (47). Treating aged mice with 4-NQO increased oral dysplasia that was associate with increased MDSCs and Tregs in tongue lesions that was in part dependent on dectin-1, a surface pattern receptor involved in fungal immunity (48). Although Wen et al. suggested that granulocytic MDSCs mediated local immune suppression, the direct lineage of these MDSCs remains unclear. Therefore, MDSCs represent another immunosuppressive mechanism to facilitate the progression of OPLs.

Other myeloid and/or granulocytic cell types may have lesser appreciated roles for OPL progression. The inflammatory microenvironment evoked by neutrophils may facilitate OPL progression. Elevated neutrophil-to-lymphocyte ratios have been associated with poor survival in multiple head and neck cancer subsites including OSCC conferring a 1.56-fold worse survival (49). Furthermore, OSCCs displayed significantly elevated neutrophil infiltration and TNFα in patients’ saliva (50). Chadwick et al. demonstrated that TNFα was necessary for OPL formation and progression in a 4-NQO model of oral carcinogenesis (50). OPLs displayed increased neutrophil infiltration that was lost with TNFα blockade. Furthermore, eosinophils infiltration was elevated in OSCC compared to dysplastic lesions suggesting that eosinophils may be necessary for malignant transformation (51). Similarly, both mast cells and eosinophil infiltration were increasingly elevated during the progression from normal epithelium to dysplasia to invasive cancer (52–55). Currently, it remains unclear how other granulocytic cells alter the tumor microenvironment to facilitate the development pre neoplastic lesions and their transition to invasive cancer. Hydroxy radical species produced by granulocytes and myeloid cells has been shown to cause DNA damage *in vitro*, which may enhance the number of genomic lesions necessary for OPLs to become cancer (56). Furthermore, neutrophils and/or other granulocytic cells can induce immune suppression in various cancers (57, 58). Therefore, the myeloid and granulocytic cell populations may contribute to OPL progression by both promoting the genetic changes necessary for malignant transformation as well as directly suppressing cytotoxic T cells that would otherwise clear abnormal cells.

## Regulation of the Immune Microenvironment by Genomic Changes in OPLs

**Figure 2:**
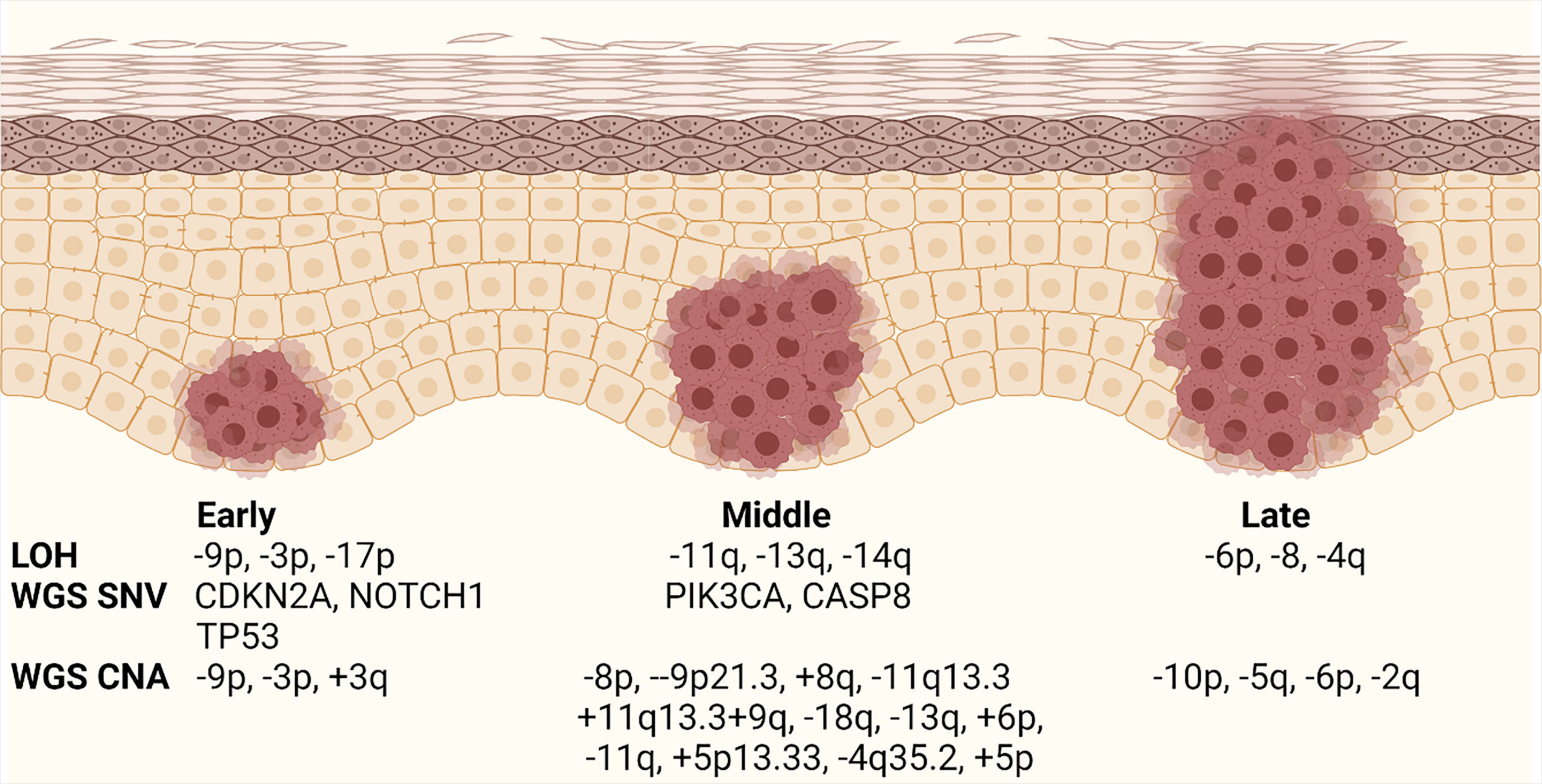
Current OPL progression model. Schematic diagram showing the relative timing of genomic events during OPL progression. The stages are labeled early, middle, and late because the data are not well associated with specific histology. LOH, loss of hetorozygosity; WGS, whole genome sequencing; SNV, single nucleotide variant; CNA, copy number alteration. Modified from Califano and Gerstung. Created with BioRender.com.

OSCC progression model describes the step-wise evolution of normal epithelium to hyperplasia to dysplasia to cancer (**Figure 2**). Large scale profiling has identified the most common genomic alterations that occur in OSCC (59), but modern genomic tools have not been used to understand the detailed evolution of those alterations during OPL progression. Early studies in OPL lesions examined loss of heterozygosity (LOH) at 10 microsatellite markers by PCR analysis and identified 9p (CDKN2A), 3p, and 17p (TP53) loss as the earliest events and 11q, 13q (RB1), 14q loss and others as relatively later events (**Figure 2**) (60). This general order of events was also identified in recent whole genome sequencing analyses of bulk HNSCC tumor samples that mapped the evolutionary history of the tumors (5). These studies identified many of the most common genomic alterations in OSCC (TP53 mutant, CDKN2A mutant/LOH, NOTCH1 mutant) as early events that can be detected in OPL.

Early studies of the genomic landscape of HNSCC led by our group and others and confirmed by The Cancer Genome Atlas (TCGA), demonstrated that mutations and deletions of the *TP53* and *CDKN2A* are the tumor suppressor genes most frequently altered somatically in HNSCC, as *TP53* and *CDKN2A* alterations are seen in up to 85% and 58% respectively of non-human papilloma virus associated (HPV negative) HNSCC (61–63). This highlights the importance of *TP53* and *CDKN2A* alterations are the most frequent genetic events occurring in the early stage of HNSCC development (63).

Another important concept to understand regarding to the early genomic alterations in OPL is field cancerization (64). Field cancerization is where genomic alterations are present throughout regions of histologically normal epithelium. These abnormal epithelial cells may not be macroscopically visible and only detectable under a microscope. In these cases, HNSCC may appear to occur *de novo*. Alternatively, patches of abnormal epithelium, which can occur in individuals with OPL lesions such as leukoplakia or erythroplakia, with macroscopically detectable lesions. The concept of field cancerization also helps describe the multicentric nature of HNSCC that either frequently recur after complete excision or new primaries that arise along the respiratory tract epithelium. may occur over time. as abnormal epithelial cells may not be.

LOH and TP53 mutations have been detected in histologically normal oral epithelium and demonstrate that field cancerization can occur with OPL and OSCC. NOTCH1 mutations are frequently detected in histologically normal sun exposed skin epithelium. Because of the similarities between cutaneous SCC mutation profiles and the biology of squamous epithelium, it is likely NOTCH1 mutations will also be detected in tobacco exposed oral epithelium. The sum total of these mutations is to drive abnormal cellular proliferation and invasion by disarming the self-destruct signals that are activated by uncontrolled proliferation. Unfortunately, there are still many gaps in our knowledge about how these genomic alterations drive OPL progression.

Transcriptomic analysis demonstrated the possibility to select for gene expression profiles of OPL more prone to malignant transformation. Saintigny et al. used gene expression microarrays to identify a 29 gene signature that predicted the progression of OPL to invasive cancer. However, many of these genes were not canonical drivers of carcinogenesis suggesting that this gene signature reflected changes of genes expression that did not likely cause malignant transformation. Sathasivam et al. used a 42 targeted gene Nanostring panel to identify an 11 gene expression signature associated with malignant progression (65). Importantly, this signature employed genes commonly altered during HNSCC carcinogenesis including TP53, NOTCH1 and CDKN2A which increases the likelihood of this signature being robust across multiple cohorts. To this end, this signature was validated using an external OPL dataset with a Hazard Ratio of 2.3. Overall, transcriptomic changes likely reflect the malignant phenotypes that arise in OPLs during malignant progression.

**Figure 3:**
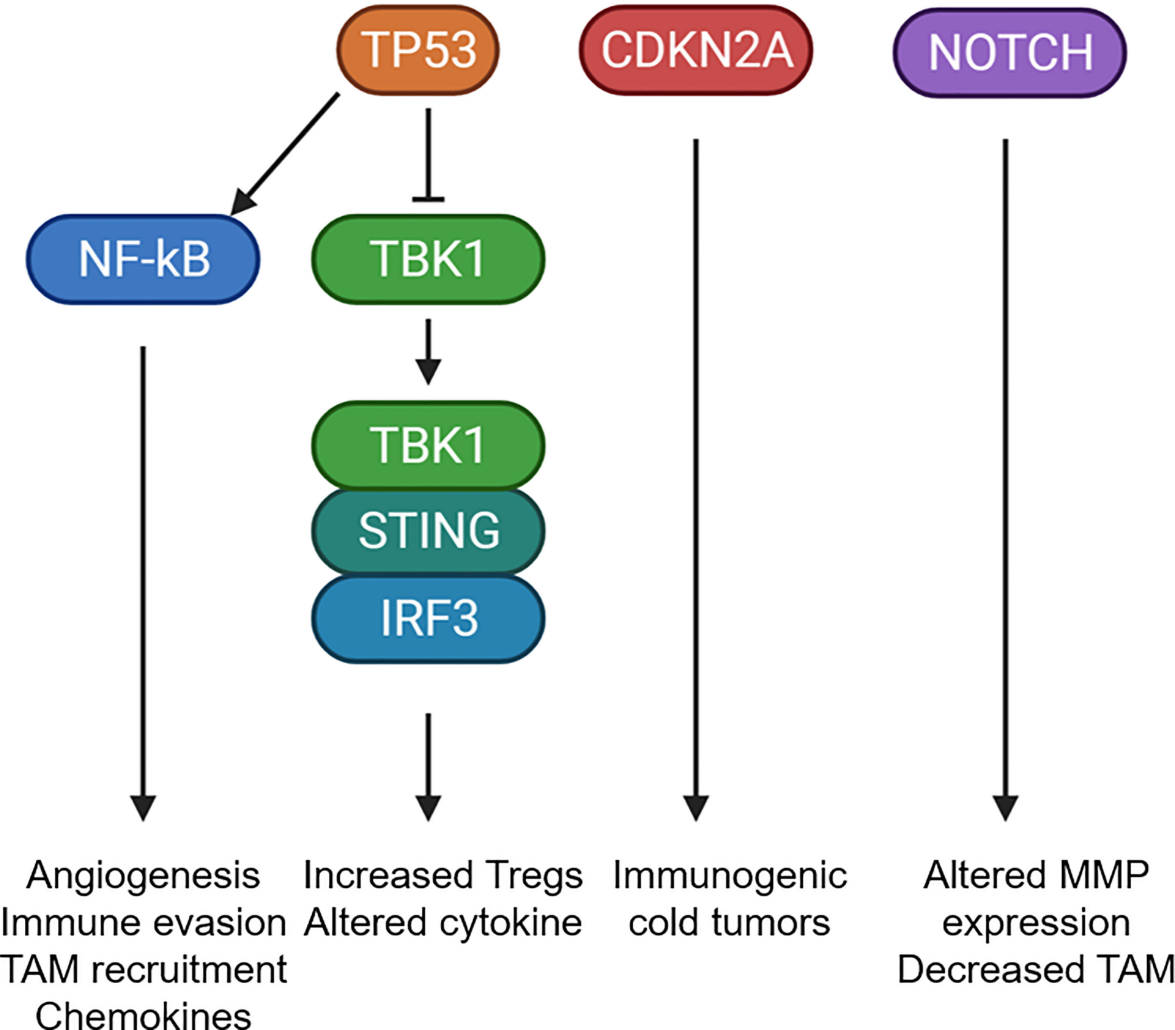
Mutational changes that potentially alter the tumor microenvironment. Schematic diagram showing the common mutational changes in OPL can impact the immune microenvironment. TP53 mutants can activate NF-kB or inhibit the STING pathway to alter cytokine expression, increase TAM recruitment, increate Treg recruitment. CDKN2A mutants has been associated with immunogenic cold tumors through currently unknown mechanisms. NOTCH1 mutants was associated with altered MMP expression and decreased TAM infiltrations. Created with BioRender.com.

The genetic changes occurring in OPL may also dictate immune microenvironmental changes that promote malignant progression. Using immune cell deconvolution of gene expression datasets from OPL, 2 different OPL subtypes were identified: (1) an immune subtype with increased T cell and immune cell infiltrate and (2) a classical subtype with LOH at 3p14, 17p13 and TP53. However, the progression to invasive cancer was not known in these subtypes (10). One study using multiplex immunofluorescence in 188 OPL patients found that OPL with high risk LOH displayed increase epithelial PD-L1 expression and increased TAM PD-L1 expression. Furthermore, PD-L1 expression was associated with increased cancer progression (66). Therefore, global genomic changes that drive OPL progression also likely dictate immunosuppressive changes in the microenvironment that are necessary for malignant progression. In addition to chromosomal changes associated with the immune microenvironment, specific genes, which are commonly mutated in OSCCs, have been implicated in dictating immune changes in OPLs and invasive cancers. The major drivers of OSCC and their impact on the immune microenvironment are detailed below (**Figure 3**):

### TP53

Studies have shown that *TP53* alterations result in biallelic loss of wild-type *TP53* function. These alterations can include mutations in one or both alleles, mutation of one allele and deletion of the other allele, mutation of one allele conferring a dominant-negative impact on the wild-type allele, or mutations which provide new functions to TP53, termed “gain-of-function” (GOF). The GOF properties impact cellular processes including proliferation, metabolism, invasion, metastasis, inflammation, drug resistance, and survival through transcription-dependent or -independent mechanisms (63, 67–74). Different studies have shown that *TP53* is frequently mutated in oral premalignant lesions and is associated with progression to invasive carcinoma (75–78). Premalignant epithelial cells expressing mutant TP53 increased the production and secretion of inflammatory mediators. Mutant p53 mice exposed to dextran sulfate sodium developed severe chronic inflammation and persistent tissue damage. These mice displayed a rapid onset of dysplastic lesions that progress to invasive carcinoma with an increased NFκB activation compared to wild-type mice, recapitulating features observed in human colitis-associated colorectal cancer (79). Furthermore, TP53 also mediated an immune escape mechanism for dysplastic lesions by recruiting tumor associated macrophages, which maintained the immunosuppressive state within the tumor microenvironment (80). In this model, GOF mutant TP53 correlated with increased iNOS, NF-κB activation leading the increased production of multiple cytokines including IL-6, CXCL5, TNF-α and CCL2. Mutant TP53 also directly interacts with NF-κB to modulate the diverse transcriptional regulators in response to chronic immune signaling (81). These biological mediators generated an inflammatory microenvironment that further increased cell survival of the transformed cells, as well as promoted angiogenesis and evasion of protective immune responses (82).

Other studies have demonstrated that loss of p53 and cooperation of KRAS in cancer cells can modulate the tumor-immune microenvironment to avoid immune destruction. Inactivation of p53 promotes the infiltration of suppressive myeloid CD11b+ cells and Tregs with an increased expression of CCR2-associated chemokines and macrophage colony-stimulating factor (M-CSF), leading to attenuated T cell responses (83). A recent study, demonstrates that TP53 missense mutations generates immune-excluded tumor microenvironments in pancreatic ductal adenocarcinoma (PDCA) mouse model, these findings correlate with clinical data in PDAC patients with a poor survival outcome (84). Since p53 plays an important role in modulating the tumor immune microenvironment, p53 mutations in OPLs suggest an important immunosuppressive role to evade immune rejection.

Recently, it has been reported that mutant p53 interferes with the cGAS-STING signaling pathway a cytoplasmic DNA sensing machinery that activates the innate immune response. Only mutant p53, binds to TANK-binding protein kinase 1 (TBK1) and prevents the protein complex between TBK1, STING, and IRF3, which is required for the transcriptional activation of IRF3. This innate immune signaling alteration by mutant p53 alters cytokine production, resulting in immune evasion (85). We have characterized the 4-Nitroquinoline 1-oxyde (4NQO) oral carcinogenesis in C57BL/6 mouse model to study the role of mutant p53 in the alteration of immune infiltrates at different stages of oral cancer. This carcinogenesis model exhibits similar histological, molecular and chromosomal alterations as observed in human oral carcinogenesis (86, 87). We and others have recently reported that 4NQO induced oral lesions expressing mutant *Trp53*-R172H contain a higher infiltration of FoxP3+ T regulatory cells (Tregs) compared to *Trp53* wild-type mice (88, 89). It is known that Tregs are suppressors of antitumor responses that disrupt the maturation of dendritic cells (DC) and prevent the activation of CD4^+^ effector and CD8^+^ cytotoxic cells in the tumor microenvironment (90). This strongly indicates that the oncogenic activity of Trp53 influence the environment to promote a higher infiltration of immune suppressor cells not only at early stages but also are detected in invasive carcinoma. Furthermore, we detected that the protein levels of STING were significantly lower in OPLs expressing mutant Trp53-R172H compared with wild-type Trp53. In addition, we observed a significant reduction of infiltrated DC cells in OPLs expressing mutant p53 (88). While infiltrating immune cells retain wild-type p53 and have normal STING, mutant p53 OPLs have decreased immune cell infiltration and may not compensate for reduced STING expression in lesions with mutant TP53. Thus, OPLs with an altered cGAS-STING signaling will prevent the secretion of type I interferons (IFN), which are induced early during tumor development (91, 92). IFNs activate dendritic cells (DCs) and promote induction of adaptive CD4^+^ and CD8^+^ T-cell antitumor immune responses (93).

These studies indicate that early genomic alterations in the *Trp53* gene of oral epithelial cells promote immunosuppressive pathways that disrupt antitumor immunity mechanisms, preventing the activation of innate and adaptive immune response and leading to high-grade lesions promoting oral neoplastic progression.

### CDKN2A

CDKN2A controls the cell cycle by inhibiting the ability of cyclin D-CDK 4/6 to phosphorylate the retinoblastoma protein (pRb) (94). pRb phosphorylation by the cyclin-CDK 4/6 leads to the dissociation of the pRb/E2F complex and progression of the cell into S phase (95, 96). The release of E2F activates CDKN2A transcription, as CDKN2A levels increase, its binding to CDK4 and CDK6 increases, inhibiting the kinase activity of cyclin D-CDK 4/6 (97). CDKN2A has been classified as a tumor suppressor, methylation studies have detected promoter hypermethylation of *CDKN2A* in oral and oropharyngeal cancer tissue as well in OPL; therefore *CDKN2A* inactivation is in part due to promoter methylation (98–102). Recently, The Cancer Genome Atlas (TCGA) data shows that 57% of HPV-negative HNSCC contains a mutation or loss of the CDKN2A gene (59), this demonstrates that additional genomic alterations on CDKN2A others than methylation are involved at early events of oral cancer development.

Other studies indicate that loss of *CDKN2A* significantly correlates with immune deserts, defined by a profile of 394 immune transcripts (103). These pieces of evidence suggest that low *CDKN2A* expression both impacts the number and the activity of the intratumoral immune cells. Additionally, some tumors lose *CDKN2A* expression as a result of the deletion of chromosome 9p21 locus. Interestingly, the deletion of adjacent genes including the α and β interferon cluster have been linked with decreased infiltration of immune cells and decreased cGAS-STING signaling in melanoma (104, 105). Recently, immunogenic analysis of clinical specimens from TCGA study and immune checkpoint trials across various cancer types and demonstrates 9p21 loss as a ubiquitous genomic correlate of the “cold” tumor-immune phenotype and primary resistance to immune checkpoint therapy (106, 107). Recently, *Cdkn2a* null mice exposed with 4NQO developed faster and more pronounced oral lesions compared to control mice; and proliferation of tumor cells with *Cdkn2a* gene deletion was associated with the progression of OSCC in mice (108). More studies are necessary in this mouse models to confirm the role of Cdkn2a in the immune surveillance mechanism of OPLs.

Yet, little is known about the interplay of mutant *p53* and inactivation *Cdkn2a* genes in the immune evasion mechanism in OPLs that evolves into tumor progression and invasion. An interesting study involving double mutant mice revealed that a combined *p53* gain of function and *Cdkn2a* inactivation generates a more aggressive skin cancer phenotype with a shorter survival and was associated to metastasis compared to single mutant mice (109). Furthermore, Cdkn2a suppresses the oncogenic activity of mutant p53 that promotes malignant progression in squamous cell carcinoma. In the same study, they analyzed HNSCC HPV-negative patients with co-occurring gain of function p53 mutations and CDKN2A homozygous deletions. Here, the survival of patients was much shorter than that of patients with tumors in which p53 mutations did not contain CDKN2A homozygous deletions, or that of patients with tumors in which homozygous CDKN2A deletions co-existed with loss-of-function mutations in p53 (109). We speculate that co-occurrence of the genomic alterations in *TP53* and *CDKN2A* in OPLs might have a worst outcome and higher probability to develop into invasive carcinoma.

### NOTCH

Another potential mutation that also alters the tumor microenvironment is loss or mutation of the NOTCH1 oncogene, which is mutated in 19% of head and neck cancers and regulates macrophage recruitment and M1/M2 macrophage polarization (59). Copy number alterations in NOTCH1 have been observed during the transition of premalignant lesions to invasive disease (110). Notch1 loss induces the expression of matrix metalloproteinases, cytokines and chemokines to alter the tumor microenvironment (111). In multiple cancers, Notch family member expression was associated increased infiltration of CD4^+^ T cells, macrophages, neutrophils and dendritic cells (112, 113). Similarly, we have observed that NOTCH1 loss in an autochthonous model of oral pre-malignant lesions alters the cytokines/chemokines driving immune cell infiltrate and correlates with loss of TAM infiltration. By contrast, Notch signaling in immune cells also dictates the extent of tumorigenic versus tumoricidal immune responses. Notch1 signaling promotes M1 TAM differentiation and inhibits MDSCs indicating that Notch1 signaling in immune cells promotes anti-tumor immunity in cancers (114–116). Consequently, global impact of NOTCH activity in OPLs is likely a competition between epithelial expression of NOTCH1 to regulate immune cell trafficking as well as myeloid NOTCH activity which determines TAM, MDSC and other immune cell phenotypes.

## Reversing Immunosuppression in OPL

While experimental studies have identified pharmacologic or molecular targeted therapies capable of reducing risk of OPL progression to cancer, there are no preventive therapeutics in routine use. Consequently, there remains an unmet need to better understand the biological and immunological features that can differentiate pre-invasive disease from invasive cancer which can also be exploited to prevent progression to malignancy.

Targeting immunosuppressive molecules may serve as an effective strategy to treat oral dysplasia and prevent malignant progression. Treatment with PD-1 blockade decreased the incidence of dysplastic lesions and invasive OSCC in a carcinogen induced 4-NOQ OPLs model (88). The Heymach group further compared several different checkpoint inhibitors including CD40, PD-1, CTLA04 and OX40, on OPL progression during oral carcinogenesis. Of these inhibitors, CD40 treatment caused the greatest reduction in OPL and OSCC tumor incidence which was associated with increased memory CD8^+^ T cells and M1 macrophage infiltration (117). Furthermore, inhibition of the adenosine receptor, A2AR, inhibited tumor growth, reduced Treg populations and increased CD8^+^ T cell infiltration in oral carcinogenesis models (27). Similarly, carotenoid or tocopherol-based treatment was associate with increased in cytotoxic lymphocytes and TAM in murine OPLs (118, 119). Activation of the IFN pathway *via* STING has also shown to inhibit the growth of HNSCC tumor models; however, expression of the STING pathway is not altered in oral dysplasia or pre-malignant lesions (120). Therefore, in pre-clinical models, checkpoint blockade, inhibition of the adenosine receptor and/or activation of the STING pathway reduced OPL incidence.

However, risk of systemic immunotherapies patients may outweigh the benefits in OPL patients that are healthy and may not develop invasive disease. One alternative to this treatment dilemma is local and controlled immunotherapy delivery to prevent oral cancer development. An ideal drug carrier should have satisfactory biocompatibility, biodegradability and controlled drug release at specific oral cavity sites. Furthermore, selecting the correct preclinical model is critical, as is designing delivery technologies that can feasibly be translated to patients. Identification of soluble inflammatory mediators produced by oral epithelial cells undergoing malignant progression which alter myeloid differentiation and/or trafficking can lead to new potential targets for therapeutic interventions.

Lately, multidomain peptide biomaterials have been developed and consist of self-assembled peptides that mimic the extracellular matrix by generating a nanofibrous network to create a hydrogel. The hydrogel can encapsulate drugs, cytokines, and growth factors and control their sustained release to permit a sustained payload release in oral cancer models (121, 122) A recent study by Shi et al. used the hydrogels loaded with PD1 immune checkpoint inhibitor to treat OPLs in p53 mutant and wild-type mice. Mice were expose to the 4NQO carcinogen, a model of carcinogenesis that represents all stages of human oral cancer. Next, hydrogels were implanted in three histological regions of the tongue to increase the ICI biodistribution. Interestingly, OPLs frequency was significantly reduced in p53 wild type mice, however high-risk OPLs were higher in mutant p53 mice (88). This study not only showed the capacity of the hydrogels to control the release of PD-1 antibody and reduce OPL frequency, but also provided evidence of the role of mutant p53 in the mechanism of immunosuppression in OPLs. Other immunoprevention studies using p53 mutant mice have showed similar results but required 8 doses of parental immunotherapy administration (89, 123), compared to a single hydrogel-PD1 dose (88). A recent comprehensive study of patient samples of leukoplakia identified that proliferative leukoplakia predicts a high rate of malignant transformation within 5 years of diagnosis. Interestingly, CD8^+^ T cell and Treg signatures with PD-L1 overexpression provides a justified approach to use anti-PD1 as immunoprevention approach in oral leukoplakia (124). Since these hydrogels are topically applied similar to TLR agonists used in melanoma, this platform provides an approach to incorporate additional immune agonists alone or to be used to increase the efficacy of checkpoint blockade. Therefore, our studies in selecting the precise preclinical mouse model was critical, and the use of hydrogel loaded with immunotherapeutic antibodies are feasible for translation immunoprevention studies.

Currently, there are only a few ongoing clinical trials studying checkpoint blockade in OPL. All of the studies use agents targeting the PD-1/PD-L1 axis including nivolumab (NCT03692325), sintilimab (NCT04065737) and avelumab (NCT04504552). Of note, these trials use both clinical and/or molecular criteria to select for patients at higher risk for progression. In these trials, clinical features such as multifocality, higher grade and/or size and/or genomic features such as LOH at 3p14 and/or 9p21 are included. Currently, these trials will add value to the role of systemic checkpoint blockade in OPL and the mechanisms for immune escape. Furthermore, there is a need to address additional immunotherapeutics that alter macrophage and/or Treg function. These studies may discover new agents for a disease with in limited therapeutic options (125).

## Discussion

OPL acquire mutations that drive the transformation of normal epithelium to invasive OSCCs, a disease to which patients frequently succumb. These mutations likely alter the immune microenvironment to suppress TILs that would otherwise potentially clear pre-malignant and malignant cells. Mutations in classical HNSCC drivers including TP53, CDKN2A and NOTCH1 have been associated with an altered an immunosuppressive tumor microenvironment. These mutations likely induce Treg and MDSC infiltration as well as the phenotypic switch of M1 TAMs to M2 TAM to suppress cytotoxic T lymphocytes. Modulation of these immunosuppressive signals using checkpoint inhibitors, targeting TAM phagocytosis with CD47 inhibitors and/or altering inflammatory pathways involving adenosine or STING may promote a tumoricidal microenvironment to activate cytotoxic lymphocytes that clear the malignant cells.

## Author Contributions

All authors contributed to the conception, design, writing and editing of this manuscript. All authors contributed to the article and approved the submitted version.

## Funding

NIH/NIDCR R01DE027445-01 (MS); NIH/NIDCR/NCI U01DE028233 (AS).

## Conflict of Interest

The authors declare that the research was conducted in the absence of any commercial or financial relationships that could be construed as a potential conflict of interest.

## Publisher’s Note

All claims expressed in this article are solely those of the authors and do not necessarily represent those of their affiliated organizations, or those of the publisher, the editors and the reviewers. Any product that may be evaluated in this article, or claim that may be made by its manufacturer, is not guaranteed or endorsed by the publisher.
